# Qualitative accounts of community health workers providing and shaping mental health support in climate-vulnerable communities in Bangladesh

**DOI:** 10.1038/s41598-026-65679-y

**Published:** 2026-08-08

**Authors:** Kyra Lilier, Erica Breuer, Samiya A. Selim, Mahmuda Akter, Rainer Sauerborn, Kate Bärnighausen

**Affiliations:** 1 Heidelberg Institute of Global Health (HIGH), Faculty of Medicine and University Hospital, Heidelberg University, 69120 Heidelberg, Germany; 2 School of Medicine and Public Health, University of Newcastle, Newcastle, 2308 Australia; 3 Centre for Sustainable Development, University of Liberal Arts (ULAB), Dhaka, 1209 Bangladesh; 4 Plan International, Dhaka, 1212 Bangladesh

**Keywords:** Community health workers, Global mental health, Community mental health support, Psychological first aid, Stigma, Qualitative research, Environmental studies, Health care, Health humanities, Psychology, Psychology, Social policy

## Abstract

Despite a clear need for mental health services in Bangladesh, there is limited access and provision, especially in areas vulnerable to the effects of climate change. Community health workers (CHWs) are commonplace primary care givers in rural communities and promise to deliver more culturally sensitive and pragmatic services. This study seeks to understand the experiences and perceptions of CHWs who have received basic training in mental health and are providing initial mental health support as the only providers of such care in underserved communities in Bangladesh. We conducted an exploratory qualitative study using in-depth interviews with 16 CHWs and one focus group discussion. Using Reflexive Thematic Analysis, we explored how CHWs introduced the stigmatized topic of mental health and balanced it with local beliefs. The training shifted CHWs’ perceptions of mental health, and they adopted strategies to reduce stigma and build trust, such as using culturally appropriate language. Their understanding of community norms allowed them to act as bridges between local and formal mental health perspectives. CHWs shape and redefine the service they deliver through their unique positioning, which makes interventions more context-relevant and appropriate. We recommend strengthening the services CHWs’ provide through continued training, material support, and formal recognition.

**Supplementary Information:**

The online version contains supplementary material available at 10.1038/s41598-026-65679-y.

## Introduction

Poor mental health is recognized as a major global health issue and remains among the ten leading causes of disease burden worldwide^1^. Meeting the rising need for mental health support and treatment remains a major challenge, particularly in low- and middle-income countries (LMICs)^2^. At the same time, scholars within the field of global mental health have questioned the applicability of universal, biomedical, and predominantly Western approaches to addressing these needs^3–6^. These critiques extend to dominant conceptualizations of mental health, including the WHO definition, which has been challenged for its Western-centric orientation, cultural inappropriateness in some contexts, and tendency to medicalize and individualize experiences of distress^7–9^. We echo these considerations and use the term ‘mental health’ more in line with the definition of Galderisi et al. 2015 (p.231 f.) as “a dynamic state of internal equilibrium which enables individuals to use their abilities in harmony with universal values of society”^10^.

Communities that are particularly vulnerable to mental distress are often the most underserved^11^. This imbalance is accelerated by climate change and changes in the social and natural environment with direct and indirect effects on mental health, such as depression, increased suicide rates, or withdrawal^12,13^. In Bangladesh, mental health services and research following the tenets of the WHO definition are limited, with formal – as in institutionalized – mental health services existing only in major cities and being almost non-existent in rural, poor communities^14,15^ and stigma towards mental illness being pervasive^16^. 18,7% (about 31 million people of a population of nearly 170 million) of Bangladeshis are considered to have a ‘psychiatric disorder’ such as depression or anxiety^17^. However there are only 260 registered psychiatrists, and less than 0.5% of national health expenditure is allocated to mental health^14,18,19^. Local mental health concepts in Bangladesh are scarcely researched or described, but the limited evidence there is reflects fundamentally different frameworks from Western psychiatric models across explanatory systems, intervention approaches, and epistemological foundations^20,21^. Supernatural and spiritual explanations and deprivation in different areas of life are considered to be the root causes of mental ill health, and community-based, collective help-seeking are modes of healing^21–23^. Additionally, concepts and terms used show incompatibilities with Western approaches^21–23^.

Community-based mental health services in underserved areas can be an essential support for common mental disorders^14,15,18^ and community-based support delivered by community health workers (CHWs) has been shown to be an effective way to deliver health services^11^. Community services promise to be most relevant for and adapted to the local context and culture, thereby being needs-driven and sensitive to local beliefs and understandings^24^. Community tailored services increase acceptance among communities and leads to more effective and efficient service delivery^11^. In addition, increased feasibility and cost-effectiveness of community services are important considerations when designing health care interventions^25^. Such services often use effective talk-based therapies but are resource-poor, delegated to lay-counselling approaches, and provided by health workers without a clinical degree^25^. This common and often a-priori model of ‘task-shifting’ means that service provision can be altered from the initial conception or model of care envisioned by clinical practitioners, as community services operate and are delivered in ways that are fundamentally redefined by the context, available resources, and training^26^. Through shared roles and responsibilities, lay mental health services shape understandings and definitions of mental health beyond biomedical frameworks^26^ and can be especially relevant in places where concepts of mental distress, its acceptability, diagnosis and treatment are defined differently or less recognized as support-needing and coincide with additional environmental stressors as a result of climate change. In Bangladesh, community health services play an important role in the pluralistic health system and often act as the first touchpoint for people in need in underserved areas^27^. However, as in many LMICs, Bangladeshi community health services have mainly focused on the acute physical health needs of the community, leaving a dearth of research regarding the type, availability, acceptability, conceptualization, and access to mental health services^28^.

To address this gap, we sought to understand the experiences and perceptions of CHWs who have received basic training in mental health and are providing mental health support in underserved communities in Bangladesh. We aim to provide insights into how CHWs navigate local understandings and stigma in relation to mental health and describe how they redefine care provision and link communities to services.

## Methods

### Study setting and design

We conducted an exploratory qualitative study using semi-structured in-depth interviews (IDIs) and a focus group discussion (FGD) in Gabura and Mongla, Bangladesh. This study was part of a larger formative qualitative study aiming to develop an intervention to support CHWs in delivering mental health support, with the plan to interview community members as the beneficiaries at a later point. Gabura is situated in the Shyamnagar Upazila of Satkhira District and lies adjacent to the Bay of Bengal, bordered by the Kholpetua and Kapatakkha rivers^29^. This area has a population of about 38,825, covering an area measuring 33 square kilometers. The economy is largely dependent on fishing and agriculture, but these sectors are increasingly challenged by climate change including salinity intrusion as well as other forms of environmental degradation^29^. Gabura’s tropical monsoon climate with its high temperature levels as well as frequent storms aggravate such vulnerabilities^30^. Cyclone Sidr (2007), Cyclone Aila (2009) and Cyclone Yaas (2021) among other natural disasters, impact socio-economic aspects and life sustenance systems and cause a rise of waterborne diseases and mental health conditions^31^.

Mongla, located in the southwestern coastal area of Bangladesh, serves as a vital port town on the Pussur River, facilitating access to the Sundarbans and Bay of Bengal^32^. It has a total area of 1461.20 square kilometers, mostly covered by the Sundarbans Forest, while 378.2 square kilometers accommodate 19 different industries in Mongla^33^. Various crises such as scarcity of water, environmental degradation, and extreme weather events, alongside social inequality, shape the lives of residents^34,35^. Agriculture and shrimp farming as well as subsistence farming are among the main sources of livelihood; however, these endeavors have been greatly hampered by soil salinity, especially during cyclones, unpredictable rainfall patterns and water scarcity^36,37^.

Both regions experience high poverty rates, high population density, and low education rates^29,37^. Residents experience cyclones, river erosion, floods, unpredictable rainfall, and drought, and it is predicted that global warming will increase the frequency and intensity of these events^30,38,39^.

### Role of CHWs in Bangladesh

CHWs are important providers of care in the Bangladeshi health care system^40^ and serve a wide range of areas that would otherwise not have access to health care services^40^. In a health care system with limited resources and a large population, feasibility is a major argument that promotes the use of CHWs^25^. CHWs are more cost-effective and time-effective in terms of education^25^. Additionally, CHWs are often local and have access to populations that could otherwise not be reached, either because of remoteness or educational, language, or cultural barriers^25^.

### SAJIDA Foundation

The SAJIDA Foundation is an NGO based in Bangladesh that has developed training for CHWs in basic mental health support in a Mental Health Pilot Project. The project operates in eight locations, covering sixty-four communities and benefiting over 3800 adults. At time of writing, the project provides community-based mental health services to underserved communities most affected by climate change. SAJIDA Foundation employs a community-based Mental Health Intervention Framework with a multi-tiered approach, utilizing CHWs for mental health promotion, basic mental health support, screening, and referrals and program officers of mental health for low-intensity counseling and case management.

The CHWs are educated to a secondary degree at minimum and receive a two-day mental health first aid training with four hours vulnerability assessment training, four hours orientation session on their role and responsibilities, compassionate listening, paraphrasing, and summarizing. They provide mental health first aid, which supports distressed people in or after a crisis event following the principle of look (identifying people in need and ensuring safety), listen (calming people and identifying needs) and link (connecting people to services or social support, facilitating coping)^41^. Alongside, CHWs identify vulnerable households by conducting a vulnerability assessment using a 10-item scale, raise awareness on mental health and share information (e.g., symptoms of mental distress and how to do self-care) for mental health promotion and prevention, and follow up with vulnerable households on a regular basis. If needed, they refer the person to the program officer holding a Higher Secondary Certificate in community mental health care for screening and intervention, who can further refer to a tele-counselling service with a psychiatrist. CHWs provide printouts and mobile phones for the telehealth service and prepare a database of vulnerable households. They are based in the villages they work in and work six hours per week, employed by SAJIDA. One CHW is responsible for 32 households. Apart from the mental health services, they deliver interventions on hygiene, livelihoods, nature-based solutions, and common physical health conditions. CHWs are supervised by the program officers and meet once a week. During supervision, they discuss the advantages and challenges encountered in their work and receive overall guidance, advice, and support.

### Sampling and recruitment

We purposively selected adult men and women (*n* = 16) for IDIs who received basic mental health training including mental health first aid by SAJIDA and were working as SAJIDA CHWs in one of the two study locations for three months to several years. The participants for the FGD (*n* = 8) were enrolled in a six-month mental health training program by SAJIDA and were included in the study to build further provider related understandings and experiences of delivering mental health support. The SAJIDA Foundation approached all CHWs that had received the mental health training and asked if they would like to participate in the study. Refusal to participate was not reported by SAJIDA. Interviewers and participants were not systematically matched by gender; while some interviews were conducted between individuals of the same gender, others involved interviewer–participant pairs of different genders, depending on interviewer availability and logistical considerations.

### Ethics

The study was approved by the Ethics Committee of the Medical Faculty of Heidelberg University on the 10th of October 2022 (S-609/2022), and by the SAJIDA Foundation on the 30th of April 2023 (2023-001-SFIRB). The study was conducted in accordance with the Declaration of Helsinki. All participants received an information sheet and provided written informed consent.

### Data collection

Prior to data collection, we trained research assistants (RAs) in qualitative data collection, where we discussed constructivism to build reflexivity among the RAs. From the 17th to the 22nd of October 2023, two Bangladeshi RAs, a woman and a man, conducted and audio-recorded 16 semi-structured IDIs and one FGD with eight CHWs. RAs were Bangladeshi postgraduate students and were fluent in English and Bangla.

The RAs gathered data using standardized instruments, including a participant information sheet with COVID-19 safety information, an informed consent form, and an interview guide available in both Bangla and English (see Supplementary Material: Interview Guides). Cover sheets captured socio-demographic data (gender, age, employment status, children, relationship status, and religion) as well as reflexive and observational notes made by the RAs. Prior to the interviews, RAs introduced the study and built rapport. Interviews lasted about 30–45 minutes, and participants chose the interview location. The FGD was held at the SAJIDA Headquarters in Dhaka.

We asked broad, open-ended questions about CHWs’ experiences, their roles, daily work and procedures, and their thoughts and opinions about mental health and expressing and sharing of emotions of the community members. We used narrative-building questions to encourage participants to share broad personal experiences and perspectives. Interviewers used probes to explore topics in greater depth and to elicit rich, contextualized accounts.

Throughout data collection, the lead author (KL) and senior author (KB) conducted daily debriefing sessions with the RAs^42^. These discussions drew on observational notes, reflections, and verbal field reports to identify themes, refine lines of inquiry, and make minor revisions to the interview guide for subsequent interviews. RAs simultaneously transcribed and translated interviews into English^43^.

### Data analysis

Following the tenets of Reflexive Thematic Analysis, we analyzed interview transcripts, debriefing notes, and reflexive and observational notes^44^. We managed our data using NVivo 15^45^ to facilitate initial coding and organize the analysis to support the different intended outcomes, namely, the publishing of manuscripts and information of intervention prototypes^46^. As we used a different guide for the FGD and IDIs, we first analyzed the transcriptions separately and then – once we had developed our initial themes – combined analysis. Embedded in a constructionist approach^47^, the first author (KL) inductively created codes derived through both semantic and latent coding to capture descriptions of CHWs’ daily work, procedures, difficulties, and opinions, but also underlying concepts and understandings of mental health and wellbeing. KL then developed themes around the CHWs’ personal experiences and perspectives on mental health shaped through their training, their experiences and perceptions of mental health within the communities, and the strategies CHWs use to connect diverse perspectives to deliver the intervention. The themes capture the narratives and conceptualization of mental health by CHWs and how they translate their understandings to the communities they work in whilst navigating local concepts, thereby shaping service delivery and addressing stigma.

Themes were discussed and refined with the lead investigators (SS& KB) and SAJIDA staff members^48^ to encourage reflexivity and consider alternative interpretations^49^.

### Reflexivity statement

The lead author and analyst KL is a medical doctor and qualitative researcher socialized and educated in Germany. Her experience of mental health practice in Europe, travel and research in Bangladesh, engagement with critical and decolonial global mental health literature, and personal worldview influenced by constructionist philosophy have shaped her motivation to investigate culturally grounded understandings of what she calls mental health and wellbeing. Her assumptions that this understanding is not universal with implications to deconstruct diagnosing and treatment regimens^3–5,8^ were challenged by her environment of Western medical colleagues, who emphasized the need for evidence-based medicine and practice, biomedical processes underlying mental diseases, and the obligation to reduce suffering through tested practices. The intricacy of Western concepts we encountered in the understandings of Bangladeshi co-workers and researchers (including co-authors) and how this may have shaped training materials of CHWs complicated the interpretation of the local concepts we present. Literature on mental health in Bangladesh does, not regularly acknowledge conflicting concepts or describe important conceptual considerations^20,23^. The lack of alternative, clearly positioned literature was addressed by reporting where Western concepts informed the studies that we cite. Acknowledging that language and communication are defining entities of reality as conceptualized by a constructionist perspective^47^, we created research tools without imposing Western terms such as ‘mental health/illness’ and – for the lack of local language resembling this concept – tried to describe rather than name what we were interested in, hence the use of the term ‘mental wellbeing’. We used narrative-style questions to encourage participants to describe their lived experiences and the ways in which mental health was understood, expressed, and enacted in everyday life, rather than asking directly for definitions or conceptualizations of mental health. In addition, differences in educational background and familiarity with mental health terminology between the research team and CHWs informed our decision to prioritize experiential accounts over abstract conceptual discussions. We acknowledge that this approach still carries underlying conceptualizations, such as that the mind and body can be separated^21^. However, by centering CHWs’ voices, we hope to – in line with critical qualitative research^50^– contribute to a change in the way mental health is understood. We also acknowledge power imbalances enacted through this research, which comprises the lead analyst KL as a Western researcher investigating local mental health concepts in Bangladesh. We have mitigated this imbalance by co-designing and executing the study with SAJIDA and Bangladeshi researchers (including co-authors).

## Results

Our results describe the insights and perspectives on the mental health of community members and CHWs as perceived by the CHWs (see Tables 1 and 2 for participant characteristics). Based on these perspectives, we show how CHWs deliver mental health support and how they connect community members to care (see Table 3: Quotes and Supplementary material Table 1: Additional quotes).

**Table 1:** Socio-demographic characteristics of CHWs (IDIs).

Characteristic	Number (%)
Female	8 (50)
Male	8 (50)
Age group	
18–29 years	9 (56)
30–39 years	5 (31)
40–49 years	2 (13)
Occupation (other than CHW)	
Student	3 (19)
Religion	
Muslim	10 (63)
Hindu	5 (31)
Not disclosed	1 (6)
Relationship status	
Single	9 (56)
Partner, living together	2 (13)
Married	5 (31)
Number of children	
0	7 (44)
1	1 (6)
2–3	6 (38)
Not disclosed	2 (13)
Highest education level	
High school education	10 (63)
Tertiary education	6 (38)
Total	16 (100)

**Table 2:** Socio-demographic characteristics of CHWs (FGD).

Characteristic	Number (%)
Female	3 (38)
Male	5 (62)
Age group	
18–29 years	6 (75)
30–39 years	2 (25)
Occupation (other than CHW)	
NGO employee	4 (50)
Teacher	2 (25)
Businessperson	1 (13)
Religion	
Muslim	4 (50)
Hindu	4 (50)
Relationship status	
Single	3 (38)
Married	5 (62)
Number of children	
0	6 (75)
1	0 (0)
2	2 (25)
Highest education level	
Tertiary education	8 (100)
Total	8 (100)

**Table 3:** Quotes.

Q1	Mental health is the human mind which we call the soul, the consciousness. If the mind is not good, no one can be happy. (Women, 32)
Q2	After all, mental health is really important. It is as important as physical health. Mental sickness leads to worse conditions. We have learned many things about mental health out of two trainings. (Women, 43)
Q3	Before I didn’t understand, before getting this training I used to mock the word mental illness. I didn’t want to understand that man can have pain inside, and it’s a disease. I also went through stress in my family, now after getting the training, I understand what mental health really is. I can understand if a person speaks to me twice, if he/she has a problem, mental problem, I can actually understand by looking at their behavior, face and speech, I would not understand if I did not get the training. (Women, 32)
Q4	[They say:] “God is keeping me happy. Alhamdulillah. […] God has given it, and it is left in the hands of God. It would not have happened if God had not given it.” There are many people who blame it on God… (Women, 32)
Q5	People from this area, they didn’t even know that there is mental health. […] They thought that health is concerned with only physical health. But mental health or that there is a thing called mental illness, and the fact that mental health impacts people more than physical health, they had no idea about it. But after we came, now we are noticing that many people are giving importance to it. People are willing to provide information regarding people who have physical and mental illness. (Man, 27)
Q6	If you talk about mental illness, they take it negatively and get angry with us. […] Basically, the people of the village understand mental problems as crazy, but they do not know that there are many stages of mental illness, at which stage a person goes crazy and at which stage there is a chance for him to come back, they have no knowledge about that. (FGD)
Q7	Men cannot cry. Women cry when something happens. Men can’t cry so their pain stays inside. (Man, 32)
Q8	She told me about this incident and burst into grief. […] She is saying that she will be humiliated if she tells the people around her, she can’t even tell anyone. […] She said ‘I couldn’t really tell anyone, I was a little relieved by telling you, it was very painful inside, I couldn’t tell anyone.’ (Women, 23)
Q9	Because of physical illness people mostly suffer mentally. So, in our area the core problem is that we don’t have any hospital or any medical center. […] So people most of the time stay sick. If we can provide them with medical help for their physical wellbeing, they might maintain better mental health. […] Their physical wellbeing is assured which will also keep them mentally stress free. Beside this, to keep them mentally healthy, we can try to counsel them or educate them. (Man, 22)
Q10	Family stress, unemployment etc. are behind poor mental health. Family pressure can be seen as family quarrels, marriage of daughters etc. And at the root of everything is financial problems when people have money, their other problems do not create so much pressure. […] Many are in debt, which causes mental stress. There is a tendency to get addicted to drugs among the youth and they are also mentally stressed about love. (Women, 38)
Q11	Most of the crisis is about the economic crisis. […] Men go outside to work most of the time, women have to run the family, women take full responsibility of the family with children. […] Then she becomes mentally ill due to various difficulties, as she has to do everything alone. (Women, 32)
Q12	Sometimes physical illness affects people’s minds, sometimes there is noise at home, busy with children, problems arise, it puts pressure on the mind. Many women are divorced it puts pressure on the mind. (Women, 32)
Q13	Because of the heat, people […] get sunburned skin and they are also irritable because of the heat. It weakens them mentally. Excessive heat creates disturbance in their mind, which is a reason for irritable mood, lack of patience etc. […] It makes them weaker. Excessive heat hampers their productivity at work. Most of them are daily laborers. Sometimes they don’t go to work because of heat, as a result they are jobless. Then even if they are physically okay but eventually mentally, they break down. (Man, 22)
Q14	If one of the side embankments breaks the entire Gabura region is flooded, and the shrimp farmers are affected economically and hence they are under stress. This mental stress of these people later turns into physical diseases. (FDG)
Q15	They express their happiness through their behavior. For example, if they receive any good news they have a smile on their face. When we talk to them, they interact openly with us and share their good news with us. Though they don’t arrange anything to celebrate joy, their words express the happiness that they are happy and in peace. Their sadness, also the same way you can say, through their behavior. Or through their conversation, paleness and depression. When we talk to them, we understand that they are actually sad. They do express. […] They do [express mental stress], but it takes time. After talking to them for a long period. (Man, 22)
Q16	Their surroundings and their words suggest that they are happy. Then they smile and talk openly. Just listening to them, you can understand that they are fine. […] Usually they don’t tell anyone directly [about their sorrows]. But if someone wants to know, then they can know. […] Through this conversation, we can understand if they are good or not. (Man, 32)
Q17	Then go to her, addressing her as ‘Apa’ (sister), trying to be close to her, not only for one day, but for four/five days. When I go and talk to her like that, then she speaks, and thinks that it will be safe to share with me. That’s how she tells us that ‘I have problems, mental problems. (Woman, 32)
Q18	I am a boy from this area. I introduce myself ‘I am a boy from your area’, many people say ‘yes, you are my own people’. This way I can easily mix with them, I get sincerity. I think this makes my job easier. (Man, 27)
Q19	I mingle with people every day, time passes, and I am a companion of people’s happiness and sadness. (Man, 27)
Q20	These people had this demand that we call them at times, but we don’t give them any tiffin. If we give them these then maybe they would give us time and they would be more attentive. But they have improved a lot compared to earlier times. They used to complain frequently that we don’t give them anything and use their time in vain. ‘Why do you come empty handed? Other NGOs give us many things, but you people don’t. Why should we give you, our time?’ They complained a lot. Though, now they have come out of that mentality a little, still if we could give them that then […] we could have been closer to them. (Man, 27)
Q21	‘Apa? How are you? How are you feeling? Are you facing any difficulties? What do you find as problems?’ We use discourse like this. ‘What would make you feel better? Have you ever tried doing something that makes you feel better?’ (Man, 27)
Q22	Simply speaking, appropriate to the situation. […] Many beneficiaries are not so educated so I speak to them in their language. […] Talking to people like them so that they understand me and feel comfortable talking to me. […] Thus, creating a closeness with them, it makes my job easier. (Man, 28)
Q23	I have heard about her suffering, maybe there is nothing to suggest, but the way she cried, she was able to express her emotions, and she liked it. She had no one to talk to. She told me. (FGD)
Q24	The best thing is that if people share, then their pressure or stress is reduced a lot. (Man, 27)
Q25	I was able to explain something to people and help them a little, they didn’t know much about health, what is mental illness, and I also thought that mental illness means ‘crazy’. But actually, it does not, I understood it and can explain it to them. And now people talk and come forward when they see us, now they think that they can share with us. I like these things very much. (Women, 23)

### Shifts of perceptions of mental health in CHWs through training

After the training, CHWS reported a shift in their perception of mental health from skeptical, unaware, or uninterested tos understanding and appreciative . The CHWs said the training also increased their skills in handling emotional situations. The CHWs explained that they were not aware of mental health before they started their training on delivering mental health services. They reported to understand more about mental health after the training and shared what mental health meant to them. Some CHWs explained that - post training - they now regarded mental health as more important than physical health and as the basis of a happy and self-efficacious life (Table 3: Q1, Q2).

Training sessions were described as important for the expansion of the CHWs’ knowledge and skills. One CHW highlighted their ability to listen to and address community members’ mental health problems, attributing their effectiveness to the training received. Another CHW admitted their previous skepticism towards mental illness, which they had dismissed as weakness, and was now understood to be a condition that required specialist support. Participants said that the training also led to personal growth, altering their behavior and attitudes towards mental health. One participant said the training had provided them with the ability to empathize and listen to others without judgment. This change in perception allowed them to educate the community about mental health, and attempt to reduce the stigma associated with mental illness. CHWs said they continuously expanded their understandings of mental health by working with the communities through reciprocal learning (Table 3, Q3).

### Mental problems in community members are perceived as influential but underrepresented by CHWs

CHWs explained that community members’ ideas of mental health differed greatly from theirs and that stigma regarding mental states of being prevented members from openly engaging with or talking about their emotional wellbeing. CHWs said the community members were unaware of mental health, that they did not recognize mental health as health-related or as distinct from physical health, explaining that there was no direct correlation to what Western medicine conceptualizes as ‘mental health’ and no direct translation used for terms like ‘depression’. CHWs told us that many community members believed that financial stability is an indicator of or equates to overall wellbeing and failed to recognize that with or without financial stability, mental illness can be a legitimate health concern. One interviewee highlighted the community’s reliance on religious explanations for their emotional wellbeing, attributing happiness and distress to the will of God and expressing fatalistic attitudes towards mental health (Table 3, Q4).

Some CHWs emphasized that community health literacy was limited and that broad health education – including mental health – is required. However, CHWs said this was slowly changing since they had begun to explore and explain health more explicitly, with some community members beginning to acknowledge and provide information about mental health issues, without naming them as such (Table 3, Q5).

CHWs spoke of high levels of stigma associated with mental illness, and discussions about mental health were seen to provoke negative reactions and anger, with many equating it to being ‘crazy’, a socially stigmatized term. The stigmatization of mental health was thought to lead to mistrust towards the CHWs (Table 3, Q6).

Participants explained that due to fear of stigma or a lack of opportunity amidst the pressures of daily life, many community members were reluctant to share their emotions. This tendency was more pronounced among men, who were described as suppressing their emotions and avoiding discussion of their feelings to follow social conventions and to maintain focus on their work and responsibilities. Participants elaborated that men do not typically cry or openly express their pain, which was thought to lead to internalized distress. Societal norms and gender expectations were assumed to strongly influence emotional expression (Table 3, Q7).

Women, despite more emotional openness, were also described as fearing humiliation and judgment from others, making them hesitant to share their struggles (Table 3, Q8).

### CHWs perceive a wide range of influences on and expression of mental health in community members

CHWs described both positive and negative influences on community members’ mental health, including social, societal, and environmental factors, and how they expressed their emotional states non-verbally. They explained that mental health was considered interconnected with family members and social relations, physical health, financial circumstances, societal structures, and religion. CHWs described how physical illness was affecting mental health and vice versa, emphasizing that physical illness frequently led to mental distress, especially in the absence of adequate medical facilities. The lack of hospitals or medical centers in the area meant that many residents remained physically unwell, which in turn exacerbated mental distress. Improved access to medical care was suggested by participants as a means to alleviate this burden, alongside mental health counseling and education (Table 3, Q9).

CHWs identified financial and social stressors as important contributors to poor mental health. Economic difficulties, such as unemployment, debt, and the financial pressures of managing a household, were prevalent (Table 3, Q10).

Participants explained that these financial issues often led to family conflicts and distress, particularly affecting women who completed most household responsibilities while men worked outside the home. They explained differences in men and women when experiencing distress, with the majority stating that women were more burdened than men (Table 3, Q11, Q12).

CHWs said that environmental factors, such as excessive heat, also contributed to the mental health challenges of the community. They described how high temperatures caused physical discomfort and irritability, reducing productivity and leading to joblessness among laborers. This was also discussed in relation to the continual flooding of the Gabura region. Broken embankments affected shrimp farmers economically, leading to heightened mental distress, which manifested as physical ailments (Table 3, Q13, Q14).

Positive influencing factors on mental health were also mentioned, such as good relationships with family and friends. Sharing problems with family and friends was described as relieving, though not frequently done.

CHWs explained how community members did not initially talk about their mental health or emotions directly, meaning they did not name them or shared how they felt. Instead, the CHWs explained how community members expressed their emotions and tensions in different ways, e.g., by showing their emotions through their body language and facial expressions or by crying. CHWs explained how their ability to identify emotional states through behavioral cues, words used, and speech styles and patterns facilitated open discussion (Table 3, Q15, Q16).

### CHWs draw on their training and understanding of local concepts to deliver mental health support

Based on the understandings and beliefs of CHWs around mental health and their perceptions and observations in their communities, CHWs developed several strategies to introduce and deliver their services in these areas where stigma was widely reported. They did so by building trust and enabling communication that allowed them to assess and support people’s mental health. The position of the CHWs between ‘formal’ health care and the community members thus permitted them to introduce the topic of mental health in a way that was culturally appropriate and sensitive to the contextual factors that influence mental health, using a range of locally adapted and developed strategies.

The process of building trust often involved repeated visits and consistent engagement over time. CHWs initially avoided discussing mental health, focusing instead on general topics or simply spending time with individuals. Engaging in normal, everyday conversations and showing genuine, persistent interest in the lives of the community over several days were effective in making community members feel comfortable and secure in sharing their problems. This approach was described as not only facilitating a safe space for community members to communicate openly about their mental health issues but also supporting community members to feel valued and understood (Table 3, Q17).

Being known in the community and considered as a part of it also helped to build trust. Local CHWs could take advantage of their familiarity with and understanding of community habits. As part of the daily lives of community members, it was easier for them to address stigmatized topics (Table 3, Q18, Q19).

Some CHWs also explained that they built trust by blending in as commonly known NGO members. Community members were used to community workers educating the households about physical health or distributing health-related materials. CHWs employed a ‘detour strategy’, starting conversations with more expected topics such as those related to physical health, and then connecting mental wellbeing to these more accepted notions of health. This approach often included sharing small material items like biscuits or presenting themselves in a professional and approachable manner, including showing name badges and appropriate clothing and behavior. Some CHWs also spoke of body language (“always put a smile on my face” (Woman, 32), “if I eat rice and go to work, then that energy will definitely show on my face” (Man, 32)) to gain trust. Initially discussing physical health helped to break the ice and build a foundation of trust, which then facilitated more open discussions about mental health.

In some instances, CHWs explained that community members initially resisted engagement with CHWs due to unmet material expectations. They compared the CHWs’ visits with those of other NGOs that provided tangible items. Over time, as community members observed the CHWs’ consistent presence and genuine concern, this resistance decreased. However, providing small tokens or refreshments was still seen as a potential way to enhance engagement and build closer relationships (Table 3, Q20).

CHWs adapted their language to strategically avoid using terms like “mental illness”, “mental health”, or “disease” (Man, 27). Instead, they opted for simpler, less stigmatizing language, referring to general “difficulties” or states of “being good” or “being bad” (Man, 27). CHWs felt this approach supported a reduction in stigma associated with mental health discussions and made conversations more accessible and less intimidating. Addressing community members with respectful and familiar terms such as “brothers and sisters” (Woman, 32) and speaking in the local dialect and in “the way we naturally talk to people of the village” (Man, 22) were key strategies CHWs explained to build rapport and trust. These practices created a sense of familiarity and respect, making community members more comfortable and willing to engage (Table 3, Q21).

Through observing non-verbal cues such as facial expressions and types of body language CHWs could gauge the comfort level and emotional state of the community members without directly asking sensitive questions. This approach allowed CHWs to understand the underlying issues and respond appropriately while maintaining a supportive and non-intrusive presence (Table 3, Q22).

Using these strategies, CHWs reported success stories when community members felt relieved after their talk or grateful for their help or referral. They explained how the beneficiaries were less stressed or happy to be able to talk about their difficulties, especially when they had no one else to talk to (Table 3, Q23).

Sharing with others, e.g., family members about problems and distress, was encouraged by CHWs and was said to be relieving and protective to one’s mental health, as described above. Many CHWs said that, once known, they were warmly welcomed into households to discuss mental health. CHWs were proud to share that they felt appreciated and trusted within the community and were motivated by how grateful households were for their care and the impact they felt they had (Table 3, Q24, Q25).

## Discussion

We provide CHWs’ insights on how mental health is understood, enacted, and expressed within their communities. CHWs described how community members shared experiences of mental health without labelling it as such, and how this act of sharing was perceived as a relief. To address the stigmatized and – as a distinct concept – unfamiliar or otherwise defined topic of mental health, CHWs used strategies such as appropriate language use or blending into communities to reduce stigma and build trust. We highlight how the CHWs’ understandings of mental health seems to have been shaped by the training and how this also enabled them to include local beliefs and develop culturally sensitive strategies that addressed stigma, responded to local challenges, and created opportunities for mental health support.

### Conceptualizing local mental health understandings

Although our participants described no direct correlation to the Western concept of ‘mental health’, including no direct translation for terms like ‘depression’^22,23^ , their descriptions allow us to draw a picture of locally enacted concepts. Investigating these under-researched narratives aligns with recent discussions challenging universalized Western concepts of mental health and emphasizes the importance and validity of cultural and local understandings^3,21,51,52^. Though not termed as such, the widespread stigma that CHWs described indicates that there was an understanding of something that is stigmatized, and though community members might not namediseases or understand their mental wellbeing as part of their health or distinct from their physical health^21^, affected individuals were described as ‘crazy’, a pattern reported in other settings with high levels of stigma^53^. This implies that community members are aware of differences in mental states of being^53^ but thatstigma and the reluctance to talk about personal emotionss indicate that one’s mental state is intimate and not or rarely shared with others, especially not with people outside the family^54,55^. However, CHWs said that community members seemed to be relieved after sharing their distress, which resonates with research where disclosure of mental states is associated with a higher quality of life^56^.

CHWs elaborated on the factors that influenced the community members’ mental wellbeing and ways of expression. They acknowledged that emotions were conveyed through body or facial expressions, and behavior and speaking style, rather than through explicit words. In Bangladesh, emotions are thought to be transferred through actions; for example, love is demonstrated by providing materially for one’s family^57,58^. Mental states might therefore be expressed more subtly and intimately, and defined through circumstance or given by God, which aligns with widespread cultural understandings^23,59,60^. These considerations point to a different conceptualization of mental health and raise important questions of how support services should be developed and delivered. If God is ultimately seen as being responsible rather than inter- and intra-human, societal, and environmental interactions, how can mental health then be targeted by an individual? What role do behavior change, community services, and traditional healing play? Which ways other than through conversations can be used to assist communities? How can ‘embodied’ support be enacted? How can services respond to severe weather events as a result of climate change? These questions reflect broader critiques of universal approaches to mental health, which emphasize the role of social determinants and structural inequalities while favoring broad population over individual approaches^7,61,62^. CHWs as frontline providers of care can help shape services that respond to these questions. This is particularly relevant in communities responding to the impacts of climate change, where modes of service provision are yet to be established^63^ and adverse effects of climate change are considered to deteriorate mental health and wellbeing^12,64^. In Bangladesh, social determinants like gender and poverty exacerbate the already high vulnerability of vast parts of the population to extreme weather events and environmental degradation^64^. More climate stress is likely to increase the number of people requiring mental health support as coping capacities become exhausted^65^ and alter the prevalence and presentation of mental health conditions^66^.

### Advantages of CHWs in creating locally relevant mental health services

Our results demonstrate the benefits of CHWs delivering mental health services, especially when they can define mental health narratives, overcome stigma, and foster accessible and self-sustaining support networks within communities^67,68^. Rapport building and the use of relatable language allowed beneficiaries to share personal topics with a stranger that they would otherwise only share with family members^67^, which is a barrier commonly faced by health care providers ^54,55^.

CHWs can build a bridge between underserved communities and ‘formal’ health care services and can serve as a mediator in both directions^67^. CHWs can navigate through religious and cultural questions that might prevent affected people from seeking formal help while acting as an entry point to the health care system and informing formal health professionals about local cultural values and understandings^67^. This way, rather than using a ‘one size fits all’ approach, professional services can adapt to local needs and learn by doing^51^, creating reciprocal learning and a culturally sensitive and needs-driven service that could mitigate stigma^11,69^.

In settings with limited access to ‘formal’ mental health care, CHWs could facilitate basic support and psychological first aid that strengthens the often-overlooked web of community health care^67,68^. The lack of referral systems to support people requiring mental health care sets a different focus for community-based interventions. Knowledge about diagnoses and treatment becomes less valuable if there is no formal treatment available; more so if this knowledge is not rooted in local and cultural norms^4,8,9^. Instead of focusing on the *what*, attention turns to the *how*: how community members can navigate their mental and emotional wellbeing, how they can be supported in their daily lives through locally available resources, and how their wellbeing can be sustained over time ^68^. Furthermore, limited research on mental health in Bangladesh in relation to services, understandings, and articulation makes it necessary to explore these topics from the bottom-up^23,51^. This research starts to build evidence for useful, applicable, and comprehensive mental health support services that can be further developed and adapted to similar contexts.

In order for the services provided by CHWs to have the greatest impact, CHWs should be supported by access to continuous training,supervision and appropriate referral options for severe cases^25^. Attrition of CHWs and levels of distress among CHWs could be mitigated with training about self-care or through peer support in Balint-style groups^40^. Finally, it is essential to ensure the provision of adequate material support in the form of salaries, travel expenses, and working materials^40^.

### Limitations

We acknowledge that the understandings of the CHWs relating to mental health are shaped through their training and not solely built from lived experience. Their training materials contained connotations and definitions of mental health derived from Western society, which may differ from local understandings. Therefore, the mental health support delivered may introduce and operate based on concepts that are new or inappropriate for the local population and might leave less room for or even suppress local understandings, which has to be considered in efforts to promote epistemic justice research^4,70^. Our findings suggest that CHWs adapted and translated training content to local contexts, helping to connect program concepts with community perspectives. At the same time, their accounts were shaped by the training they received, and consequently, statements that mental health concepts were absent within communities should be interpreted cautiously, as they may reflect newly acquired understandings of mental health rather than the absence of local conceptualizations. Nuance and meaning of language are altered and may be lost through the introduction of terms used in the interview guide – though we addressed this issue via reflective translation and as described in the reflexivity section. In addition, the benefits and value of the service are qualitative descriptions rather than outcome measures of effectiveness.  

## Conclusion

CHWs with basic mental health training can act as a bridge between ‘formal’ mental health systems and underserved, climate-vulnerable communities. CHWs apply nuanced approaches to mental health and use locally adapted strategies to build trust and raise awareness. In low-resource settings with high levels of stigma, such approaches can enable community members to access support, and CHWs can help redefine care.

## Supplementary Information

Below is the link to the electronic supplementary material.

Supplementary Material 1

## Data Availability

The datasets generated and analyzed during the current study are available in “heiDATA”, an institutional repository for Open Research Data from Heidelberg University, 10.11588/DATA/MMUR3V.
