# Supplementary material for: Qualitative accounts of community health workers providing and shaping mental health support in climate-vulnerable communities in Bangladesh
Source: Sci Rep. 2026 Aug 8;16:24549. doi: 10.1038/s41598-026-65679-y (PMC13452806; doi:10.1038/s41598-026-65679-y)
Supplement: Supplementary file 1 [file 41598_2026_65679_MOESM1_ESM.docx]

# Supplementary material

## Table 1: Additional quotes:

| Topic | Theme(s) | Quote |
| --- | --- | --- |
| Perception of CHWs | Importance of mental health | Regarding mental health, we say that if someone's mental health is not good, if you are not in a good mood, then you feel not good to work, and turmoil will be created in the family, you cannot do any work seriously, because your mental health is not good. (Man, 28) |
|  | Changes in perceptions through training | I received training in subjects that I had no idea about and learned about them through these trainings. When I go to people as a CHW, listen to them and listen to their problems as a first aid mental health worker I can help them in some way. This is what I like the most. (Man, 32) |
|  | Changes in perceptions through training | After we received the training, there was a change in myself, my behavior changed, and the perception of our mental health improved. I listen to people and don’t make judgments. (Man, 28) |
|  | Changes in perceptions through training | I was able to explain something to people and help them a little, they didn't know much about health, what is mental illness, and I also thought that mental illness means crazy. But actually, it does not. I understand it and can explain it to them. And now people talk and come forward when they see us, they think that they can share with us. I like these things very much. (Woman, 23) |
| Perception in community members | Physical health as only aspect of health | If they are financially well off, they think that they are fine. But they don’t know what the actual state of their mind is. They think mental illness is not a disease. If there is pain in the waist and bones, then this is a disease. (Woman, 32) |
|  | Employment as an influencing factor on mental health | Unemployment is a big issue in my area; it causes mental distress. So, if we could provide employment for them, I think it could be a way out of this situation. […] Actually, being engaged in work helps keeping a mind fresh. (Man, 22) |
|  | Relationships as an influencing factor on mental health | If everyone in their family stays well then their mental health also remains healthy. […] Suppose my relationship with my father is not in good terms which implies that my father is in depression and so am I. Instead, if we have a beautiful understanding between us, with my mother, my father and with all my family members, if I can maintain a good relationship with everyone then my mental pressure will remain at the minimum. Even If I bring any mental pressure from outside and share it with them and if they support me a little then I will be able to stay better up to a point. (Man, 22) |
|  | Recognizing mental health through speech style and facial expression | She is actually crying to express her grief, or many people talk very emotionally that if you look at their speech style, you can understand that they are really in pain. … When they are happy, they talk cheerfully, joke, and smile. It means that they have a feeling that they are a little at peace or a little happy. (Man, 28) |
|  | Recognizing mental health through speech style and facial expression | When we go to people and talk to them, they get that benefit and smile and talk to us. Just by listening to their words, you can tell that they are happy, and by looking at their facial expressions, you can tell whether a person is happy or not. (Man, 18) |
|  | Recognizing mental health through speech style  Reluctance to share feelings | When a person is happy, talking to him shows that he is happy. And when a person is sad, even if you try to talk to him a thousand times, but you don't get a response, it means that he is not happy. Usually no one shares happiness and sadness except very close people but talking to them can tell whether he is in pain or happy. (Women, 38) |
|  | Reluctance to share feelings | Most people don't want to share their sadness and remain silent and sometimes they attempt suicide. (Man, 18) |
|  | Reluctance to share feelings | Men don't want to give us much time and don't want to say their feelings. Men are at work, they say, if I spend an hour and a half with you, what will happen to my work? But yes, there is a man […], he has given enough time, after talking to him he is much better now. (Women, 32) |
| Strategies | Building trust | They do not want to share this matter, I went to a house, how are you? Yes, I'm fine. That’s it for the first day. next time when we go again, they trust a little, we say that if you have any problem, mental problem, you can share with us. We will keep it a secret, we will tell you where it would be better to go and get treatment. Then they believe, then that's how they tell us their problem about their family ...everyone can have a problem. (Woman, 32) |
|  | Adapted language use | They don't understand the words ‘mental health’, ‘mental illness’. ‘Being good’, ‘being bad’, I use these things. (Man, 27) |
|  | Adapted language use | We don’t use it. There is a word called ‘Shonirvor Biva’ (This word had been taught by Sajida trainers which is the abbreviation of several words)’ which we use in the field. First, we enter a house and share greetings and ask ‘how are you’. Always give priority to them and try to get their response whether they are feeling okay to talk or not. Then […] find the perfect place where they can feel comfortable to talk. Then start the conversation and observe facial expression to get how the expression is changing with words. We don’t ask them, we try to get it by watching the face. And listen to them very attentively. After that, if there is any issue, we talk about it. We also talk about her wellbeing. (Woman, 31 |
|  | Adapted language use | We don't use the word mental illness or disease, we usually say we will talk about mental health. (Man, 32) |
|  | Adapted language use | I never use the word mental problem. We often use the word 'difficulty' when we ask what difficulties you have. (Woman, 38) |
|  | Adapted language use | When we go to a beneficiary, we talk normally so that they don’t think someone else is coming, I act like I am one of them. We talk to the people of the village the way we naturally talk to people of the village. (Man, 22) |

## Interview Guide: IDI- Community Health Worker

As we went over in the consent form, all of the information you provide will be kept confidential. Just as a reminder our interview will probably last around 45-60 minutes. Do you have any questions before we begin? May I start the recording? *[Start recording]*

*যেহেতু আমরা সম্মতি ফর্মে পূরণ করেছি, আপনার দেওয়া সমস্ত তথ্য গোপন রাখা হবে। জানিয়ে রাখি, আমাদের সাক্ষাত্কার সম্ভবত প্রায় ৪৫-৬০ মিনিট স্থায়ী হবে। আমরা শুরু করার আগে আপনার কোন প্রশ্ন আছে? আমি কি রেকর্ডিং শুরু করতে পারি? [রেকর্ডিং শুরু করুন]*

**Good [afternoon/morning] thank you for participating today**! I have asked you to meet with me in the hopes of learning more about your experience and thoughts about living here and your interactions with community health workers. Some of the questions I will ask you may not want to answer and that is fine. Remember that your answers are confidential and participation is completely voluntary. Also please keep in mind that there are no right or wrong answers, I am interested in anything you can share with me.

**শুভ [বিকেল/সকাল] আজ অংশগ্রহণ করার জন্য আপনাকে ধন্যবাদ!** কমিউনিটি হেলথ ওয়ার্কার হিসেবে কাজ করার বিষয়ে আপনার অভিজ্ঞতা এবং চিন্তাভাবনা সম্পর্কে আরও জানার আশায় আমরা আপনাকে আমাদের সাথে দেখা করতে বলেছি। আমার কোন প্রশ্ন যদি আপনি উত্তর দিতে না চান, কোনো সমস্যা নেই তাতে। মনে রাখবেন যে আপনার উত্তরগুলি গোপনীয় এবং অংশগ্রহণ সম্পূর্ণরূপে স্বেচ্ছাসেবী/ ঐচ্ছিক। এছাড়াও দয়া করে মনে রাখবেন যে কোনও সঠিক বা ভুল উত্তর নেই, আপনি আমার সাথে শেয়ার করতে পারেন এমন যেকোন কিছুতে আমি আগ্রহী।

**Questions**

**প্রশ্নাবলি**

1. To begin, please tell me a bit more about yourself.

১। শুরু করার জন্য, দয়া করে আমাকে আপনার সম্পর্কে আরও কিছু বলুন।

2. Please tell me what you think about your work in the community.

২।দয়া করে আমাকে বলুন যে এই এলাকায় আপনার কাজ সম্পর্কে আপনি কি মনে করেন?

3. Keeping in mind that there are no right or wrong answers, in your understanding, what is your role as a community health worker?

৩। মনে রাখবেন যে কোন সঠিক বা ভুল উত্তর নেই,আপনার মতে, কমিউনিটি স্বাস্থ্যকর্মী হিসাবে আপনার ভূমিকা কি?

a. Why do you do this job?

ক। আপনি কেন এই কাজ করেন?

b. What do you enjoy about your job?

খ। আপনার কাজের কোন অংশটি আপনি উপভোগ করেন?

c. What makes your job difficult?

গ। কি আপনার কাজকে কঠিন করে তোলে?

d. What makes your job easy?

ঘ। কোন বিষয়গুলো আপনার কাজকে সহজ করে তোলে?

e. What are you trying to achieve?

ঙ । আপনার এই কাজের মধ্য দিয়ে আপনি কি অর্জন করতে চেষ্টা করছেন?

4. How do you feel about the health of the people in the community?

৪।	এই এলাকার মানুষের স্বাস্থ্য সম্পর্কে আপনি কি মনে করেন?

a. What do you think makes them healthy?

ক।	আপনার মতে কোন জিনিসগুলো তাদের সুস্থটা বজায় রাখে?

b. What are the unhealthy behaviors you see?

খ।	আপনি তাদের মধ্যে কোন অস্বাস্থ্যকর আচরণ লক্ষ্য করেন?

5. Please walk me through a normal day working with the community here.

৫।	এই এলাকায় কাজ করার একটি সাধারণ দিন সম্পর্কে আমাকে জানান।

6. Please walk me through an example of when you felt you made a positive difference to a persons health.

৬। আমাকে এমন একটি উদাহরণ দিন যখন আপনি একজন ব্যক্তির স্বাস্থ্যে ইতিবাচক পরিবর্তন এনেছেন বলে মনে করেছেন।

a. What happened on that day?

ক। কি হয়েছিল সেদিন?

b. What do you think you did well?

খ।সেদিন কোন কাজটি আপনি ভাল করেছিলেন বলে মনে করেন?

c. What would you have liked to have done differently?

গ।	আপনি ভিন্নভাবে কি করতে চেয়েছিলেন?

d. What would have helped you to do your job better?

ঘ। আপনার কাজটি আরও ভালোভাবে করতে আপনাকে কী সাহায্য করবে?

7. Please walk me through an example of when you felt you did not achieve what you wanted to regarding a persons health.

৭। আমাকে এমন একটি উদাহরণ দিন যখন একজন ব্যক্তির স্বাস্থ্য সম্পর্কে আপনি যা চেয়েছিলেন তা অর্জন করতে পারেননি।

a. What happened on that day?

ক। কি হয়েছিল সেদিন?

b. What do you think you did well?

খ। সেদিন কোন কাজটি আপনি ভাল করেছিলেন বলে মনে করেন?

c. What would you have liked to have done differently?

গ।	আপনি ভিন্নভাবে কি করতে চেয়েছিলেন?

d. What would have helped you to do your job better?

ঘ। সেদিন কি হলে আপনার কাজটি আরও ভালোভাবে করতে সাহায্য করতো ?

8. Who do you think benefits the most from the work that you do?

৮। আপনি যে কাজটি করেন তা থেকে কে সবচেয়ে বেশি উপকৃত হবে বলে আপনি মনে করেন?

9. How do you think that the weather impacts the health of the community here?

৯। আপনার মতে, এখানকার আবহাওয়া কিভাবে এই সম্প্রদায়ের মানুষের স্বাস্থ্যের উপর প্রভাব ফেলে?

a. Flooding?

ক। বন্যা?

b. Drought?

খ। খরা?

c. Extreme heat?

গ। প্রচন্ড গরম?

d. Sudden onset events?

ঘ। আকস্মিক দূর্যোগেরসুত্রপাত?

e. Long term impacts?

ঙ। দীর্ঘমেয়াদী প্রভাব?

f. Other?

চ। অন্যান্য?

10. What things are in place to help people here with extreme weather events like flooding?

১০। বন্যার মতো চরম আবহাওয়া ঘটিত ঘটনা্র ক্ষেত্রে এখানে লোকেদের সাহায্য করার জন্য কী কী বিষয় রয়েছে?

a. Early warning?

ক। পূর্ব সতর্কবার্তা?

b. Post event support?

খ। দূর্যোগ পরবর্তী সাহায্য?

11. How do people talk about their mental wellbeing here?

১১। মানুষ কিভাবে তাদের মানসিক সুস্থতার কথা বলে?

a. How do they describe happiness?

ক। তারা কিভাবে সুখ বর্ণনা করে?

b. How do they describe sadness?

খ। তারা কীভাবে দুঃখের বর্ণনা দেয়?

c. How do they describe stress?

গ। তারা কিভাবে চাপ বর্ণনা করে?

12. What can you do to support the mental being of people here?

১২। এখানকার মানুষের মানসিক সত্তাকে সমর্থন করার জন্য আপনি কী করতে পারেন?

13. What other resources do you need to help people with their mental wellbeing?

১৩। মানুষের মানসিক সুস্থতার বিষয়ে সাহায্য করার ক্ষেত্রে আর কি ধরনের রিসোর্চের/ সহায়তার প্রয়োজন আছে বলে মনে করেন?

a. Professional skills?

ক। পেশাগত দক্ষতা?

b. Physical space (for meetings etc.)?

খ। শারীরিক উপস্থিতির স্থান ( মিটিং ইত্যাদির জন্য)

c. What couldn’t you do?

গ। আপনি কি করতে পারেননি?

d. What would you liked to have done better?

ঘ। আপনি কি করলে আরো ভাল করতে পারতেন?

14. What would you personally like to see or do here to help with mental wellbeing support?

১৪। মানসিক সুস্থতা সহায়তার জন্য আপনি ব্যক্তিগতভাবে এখানে কী দেখতে বা করতে চান?

15. What do you think are the main reasons for poor mental wellbeing here?

১৫। মানসিক সাস্থ্য খারাপ হওয়ার পেছনে প্রধান কারণ কি কি?

16. What do you think are the main reasons for good mental wellbeing here?

১৬। মানসিক সাস্থ্য ভালো হওয়ার পেছনে প্রধান কারণগুলো কী কী?

17. How do things like early warning systems for extreme weather events help with mental wellbeing?

১৭। চরম দুর্যোগের ঘটনাগুলির জন্য প্রাথমিক সতর্কতা ব্যবস্থার মতো বিষয়গুলি কীভাবে মানসিক সুস্থতার ক্ষেত্রে সাহায্য করে বলে মনে করেন?

a. Please give an example of something that helps with mental wellbeing in relation to weather events

ক। আবহাওয়া সংক্রান্ত ঘটনাগুলো সাথে সম্পর্কিত মানসিক সুস্থতাকে সাহায্য করে এমন কিছু উদাহরন দিন।

18. Please walk me through an example of when you have used your recent training to support someone.

১৮। আপনার সাম্প্রতিক প্রশিক্ষণ ব্যবহার অরে কাউকে সহযোগিতা করেছেন, দয়া করে এমন একটি উদাহরণ দেন।

a. What happened?

ক। কি হয়েছিল?

b. Do you think you were successful in helping them and why or why not?

খ। আপনি কি মনে করেন আপনি তাদের সাহায্য করতে সফল হয়েছেন এবং কেন হয়েছেন বা হননি?

c. What more could you do?

গ। আপনি আর কি করতে পারতেন?

d. What would you keep the same?

ঘ। আপনি কোনটা অপরিবর্তিত রাখবেন?

e. What would you change?

ঙ। আপনি কোনটা পরিবর্তন করবেন?

19. What are the differences and similarities between men and womens mental wellbeing?

১৯। পুরুষ এবং মহিলাদের মানসিক সুস্থতার মধ্যে পার্থক্য এবং মিল কি?

a. Why?

ক। কেন?

20. What do you think about the mental wellbeing of children in this community?

২০। এই সম্প্রদায়ের/ এলাকার শিশুদের মানসিক সুস্থতা সম্পর্কে আপনি কি মনে করেন?

a. Is it different or the same between girls and boys?

ক। ছেলে এবং মেয়েদের মধ্যে এটি কি ভিন্ন নাকি একই?

b. What do you think children worry about?

খ। আপনার মতে বাচ্চারা কি নিয়ে উদ্বিগ্ন?

c. What do you think makes children happy here?

গ। আপনার মতে এখানে কোন বিষয়গুলো বাচ্চাদের খুশি করে?

21. What are the words and expressions you use to talk about mental health?

২১। মানসিক স্বাস্থ্য সম্পর্কে কথা বলার জন্য আপনি কোন শব্দ এবং অভিব্যক্তিগুলি ব্যবহার করেন?

22. Would you like to add anything else? Is there a question that you think is important but that I didn’t ask?

২২। আপনি আর কিছু যোগ করতে চান? এমন কোন প্রশ্ন আছে যা আপনি গুরুত্বপূর্ণ মনে করেন কিন্তু আমি জিজ্ঞাসা করিনি?

## Interview Guide: **FGD - Community Health Worker**

As we went over in the consent form, all of the information you provide will be kept confidential. Just as a reminder our interview will probably last around 45-60 minutes. Do you have any questions before we begin? May I start the recording? *[Start recording]*

*যেহেতু আমরা সম্মতি ফর্মে পূরণ করেছি, আপনার দেওয়া সমস্ত তথ্য গোপন রাখা হবে। জানিয়ে রাখি, আমাদের সাক্ষাত্কার সম্ভবত প্রায় ৪৫-৬০ মিনিট স্থায়ী হবে। আমরা শুরু করার আগে আপনার কোন প্রশ্ন আছে? আমি কি রেকর্ডিং শুরু করতে পারি? [রেকর্ডিং শুরু করুন]*

**Good [afternoon/morning] thank you for participating today**! We have asked you to meet with us in the hopes of learning more about your experiences and thoughts about working as a community health worker. Some of the questions I will ask you may not want to answer and that is fine. Remember that your answers are confidential and participation is completely voluntary. Also please keep in mind that there are no right or wrong answers, I am interested in anything you can share with me.

**শুভ [বিকেল/সকাল] আজ অংশগ্রহণ করার জন্য আপনাকে ধন্যবাদ!** কমিউনিটি হেলথ ওয়ার্কার হিসেবে কাজ করার বিষয়ে আপনার অভিজ্ঞতা এবং চিন্তাভাবনা সম্পর্কে আরও জানার আশায় আমরা আপনাকে আমাদের সাথে দেখা করতে বলেছি। আমার কোন প্রশ্ন যদি আপনি উত্তর দিতে না চান, কোনো সমস্যা নেই তাতে। মনে রাখবেন যে আপনার উত্তরগুলি গোপনীয় এবং অংশগ্রহণ সম্পূর্ণরূপে স্বেচ্ছাসেবী/ ঐচ্ছিক। এছাড়াও দয়া করে মনে রাখবেন যে কোনও সঠিক বা ভুল উত্তর নেই, আপনি আমার সাথে শেয়ার করতে পারেন এমন যেকোন কিছুতে আমি আগ্রহী।

**Questions প্রশ্নাবলি**

1. How do you feel about the general health of the people in the community?

এই এলাকার মানুষের সাধারণ স্বাস্থ্য সম্পর্কে আপনি কি মনে করেন/ আপনার মতামত কি?

a. What do you think makes them healthy?

আপনার মতে কোন জিনিসগুলো তাদের সুস্থটা বজায় রাখে?

b. What are the unhealthy behaviors you see?

আপনি কি তাদের কোনরকম অসাস্থ্যকর আচরণ লক্ষ্য করেছেন বা দেখেছেন?

2. How do you think that the weather impacts the health of the community here?

আপনার মতে, এখানকার আবহাওয়া কিভাবে এই সম্প্রদায়ের মানুষের স্বাস্থ্যের উপর প্রভাব ফেলে?

a. What has the main impact? প্রধান প্রভাবগুলো কি?(Flooding/ Drought/ Extreme heat/ Sudden onset events/ Long term impacts/ Other )

বন্যা/ খরা/ অতিরিক্ত তাপ/ আকস্মিক প্রাকৃতিক দূযোগের সুত্রপাত/ দীর্ঘমেয়াদী প্রভাব/ অন্যান্য

b. Why and how? কেন এবং কিভাবে?

3. How do people talk about their mental wellbeing?

মানুষ কিভাবে তাদের মানসিক সুস্থতার কথা বলে?

a. How do they describe happiness? সুখ বা খুশিকে তারা কিভাবে ব্যাখ্যা/ বর্ননা করে? sukh ba khushi ke tara kibhabe bekkha kore/bornona kore?
b. How do they describe sadness? তারা কীভাবে তাদের দুঃখ/কষ্টকে
c. How do they describe stress? মানসিক চাপ/প্রেসারে তারা কিভাবে ব্যাখ্যা/ বর্ণনা করে?

4. What can you do to support the mental wellbeing of people?

মানুষের মানসিক সুস্থতা বজায় রাখতে আপনি তাদের কী ধরনের সাহায্য করতে পারেন?

5. What other resources are needed to help people with their mental wellbeing? মানুষের মানসিক সুস্থতা নিশ্চিত করতে বা এ বিষয়ে সাহায্য করার ক্ষেত্রে আর কি ধরনেরসংস্থান/বন্দবোস্ত/ব্যবস্থার প্রয়োজন আছে বলে মনে করেন?
   a. Professional skills? পেশাগত দক্ষতা?
   b. Physical space (for meetings etc.)? নির্দিষ্ট জায়গা/ স্থান (মিটিং ইত্যাদির জন্য)?
   c. What couldn’t you do? আপনি কি করতে পারেননি?
   d. What would you liked to have done better? আপনি কি করলে আরো ভাল করতে পারতেন?

6. What are the main reasons for poor mental wellbeing?

মানসিক সাস্থ্য খারাপ হওয়ার প্রধান কারণ কি কি?

7. What are the main reasons for good mental wellbeing?

মানসিক সাস্থ্য ভালো হওয়ার প্রধান কারণগুলো কী কী?

8. How do things like early warning systems or post event support for extreme weather events help with mental wellbeing?

চরম দুর্যোগের ঘটনাগুলির জন্য প্রাথমিক সতর্কতা ব্যবস্থার এবং দুর্যোগ পরবর্তী সহায়তার মতো বিষয়গুলি কীভাবে মানসিক সুস্থতার ক্ষেত্রে সাহায্য করে বলে মনে করেন?

a. Please give an example of something that helps with mental wellbeing in relation to weather events

দয়া করে এমন কিছুর উদাহরণ দিন যা আবহাওয়ার ঘটনাগুলির সাথে সম্পর্কিত মানসিক সুস্থতাকে সাহায্য করে

9. Please walk me through an example of when you have used your recent training to support someone.

আপনার সাম্প্রতিক প্রশিক্ষণ ব্যবহার অরে কাউকে সহযোগিতা করেছেন, দয়া করে এমন একটি উদাহরণ দেন।

a. What happened? কি হয়েছিল?
b. Do you think you were successful in helping them and why or why not? আপনি কি মনে করেন আপনি তাদের সাহায্য করতে সফল হয়েছেন এবং কেন হয়েছেন বা হননি?
c. What more could you do? আপনি আর কি করতে পারতেন?
d. What would you keep the same? আপনি কোনটা অপরিবর্তিত রাখবেন?
e. What would you change? আপনি কোনটা পরিবর্তন করবেন?

10. What are the differences and similarities between men and women's mental wellbeing?

পুরুষ এবং মহিলাদের মানসিক সুস্থতার মধ্যে পার্থক্য এবং মিল কি?

a. Why? কেন?

11. What do you think about the mental wellbeing of children in this community?

এই সম্প্রদায়ের/ এলাকার শিশুদের মানসিক সুস্থতা সম্পর্কে আপনি কি মনে করেন?

a. Is it different or the same between girls and boys? ছেলে এবং মেয়েদের মধ্যে এটি কি ভিন্ন নাকি একই?
b. What do you think children worry about? আপনার মতে বাচ্চারা কি নিয়ে উদ্বিগ্ন?
c. What do you think makes children happy here? আপনার মতে এখানে কোন বিষয়গুলো বাচ্চাদের খুশি করে?

12. Would you like to add anything else? Is there a question that you think is important but that I didn’t ask?

আপনি অন্য কিছু যোগ করতে চান? এমন কোন প্রশ্ন আছে যা আপনি গুরুত্বপূর্ণ মনে করেন কিন্তু আমি জিজ্ঞাসা করিনি?
